# Efficacy of Interferon α-2b and Ribavirin against *West Nile Virus* In Vitro

**DOI:** 10.3201/eid0801.010252

**Published:** 2002-01

**Authors:** John F. Anderson, James J. Rahal

**Affiliations:** * Connecticut Agricultural Experiment Station, New Haven, Connecticut, USA; † New York Hospital Queens and Weill College of Medicine, Cornell University, New York, New York, USA

To the Editor: *West Nile virus* (WNV) infected humans in the Western Hemisphere for the first time in the late summer of 1999. During 1999 and 2000, nine deaths occurred among 80 patients with meningitis or encephalitis in New York City; Westchester County, New York; New Jersey; and Connecticut (*1*–*3*). Effective antiviral agents are unknown for infections caused by WNV. Odelola (*4*) described 83% survival of WNV-infected mice and eradication of virus from brain when 1.5 mg. of ribavirin was administered by intraperitoneal injection after virus inoculation. Survival of controls was 25%. More recently, Jordan et al. have reported inhibition of WNV by a relatively high concentration of ribavirin (200 _M) given after infection of human oligodendroglial cells in vitro (*5*). Shahar et al. (*6*) reported protection of fetal mouse spinal cord tissues with mouse alpha and beta interferon before inoculation with WNV. We tested human recombinant interferon alpha-2b and ribavirin for activity against WNV in a primate cell system similar to that used to measure the effect of these agents on *Bovine viral diarrhea virus*, a cultivatable, closely related surrogate for *Hepatitis C virus*.

Vero cells were cultured at 37^o^ and 5% CO_2_ in a 96-well microtiter plate. Approximately 13,000 cells were seeded in each well 24 hours before specific concentrations of ribavirin or interferon alpha-2b were added. Approximately 2.5 X 10^3^ PFU of WNV isolated from *Culex pipiens* (*7*) was added 1.5-2 hours after or before the addition of interferon alpha-2b or ribavirin to Vero cells. Forty-four hours after treatment, a colorimetric proliferation assay was used to measure viable cells in each treated well according to the protocol of Promega (Madison, WI). Cells exposed to specific concentrations of antiviral compounds, but without WNV, were used as negative controls.

 Interferon alpha-2b was protective and therapeutic. Interferon alpha-2b inhibited viral cytotoxicity at low dosage when applied before or after infection of cells with WNV. For example, viral protection was observed from 3,000 U/mL to 188 U/mL 2 hours before infection of cells with WNV. Interferon alpha-2b was also therapeutic when applied after cells were infected with WNV. Virus-induced cytotoxicity was inhibited by concentrations of ≥5.9 U/mL when added 1.5 hours after infection (Figure). The optical density 490 values in these tests were significantly different (p<0.05, using Tukey HSD multiple comparison test) when compared with the uninfected cells.

**Figure:**
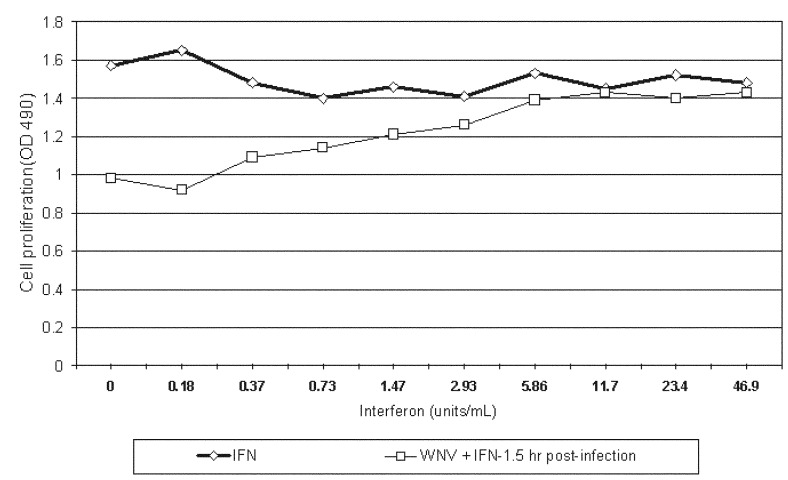
Effect of varying concentrations of interferon α-2b (FN) on *West Nile virus-*infected Vero cells. The vertical axis represents a colorimetric assay of cellular lactic dehydrogenase, which is directly proportional to cell viability and proliferation. OD = optical density.

Ribavirin was protective but not therapeutic in vitro. Cells were protected at dosages of 400 and 500 _M but not at dosages of ≤300 _M of ribavirin applied 2 hours before infection of cells with WNV. A cytotoxic effect of ribavirin occurred at concentrations of 600-1,000 _M.

In humans, daily doses of 3 million units of interferon result in serum levels of 10-20 U/mL, well above that required for in vitro efficacy (*8*). In contrast, oral ribavirin doses of 2,400 mg daily yield a steady-state serum concentration of 3-4 _g/mL after several days, approximately 12-40-fold less than the in vitro inhibitory concentrations of 200-500 _M (50-125 _g/mL) noted by Jordan et al. (*5*) and in this study. Intravenous administration of 4 g daily, as used in the treatment of Lassa fever, would be required to reach a potentially effective serum concentration (*9*,*10*). However, intracellular accumulation and phosphorylation of ribavirin may account for its therapeutic effect in mice (*4*).

We conclude that interferon alpha-2b possesses greater therapeutic activity in vitro than ribavirin, with a potentially greater therapeutic ratio in humans. Whether combination therapy, as employed against hepatitis C, may be optimal requires further study.
